# Efficacy and Safety of CKDB‐501A in Treating Moderate‐To‐Severe Glabellar Lines: A Randomized, Double‐Blind, Active‐Controlled, Multi‐Center Phase III Trial

**DOI:** 10.1111/jocd.70305

**Published:** 2025-06-30

**Authors:** Sun Young Choi, Beom Joon Kim, Yang Won Lee, Won‐Serk Kim, Yi Na Yoon, Jin Seop Kim

**Affiliations:** ^1^ Department of Dermatology Chung‐Ang University Gwangmyeong Hospital, Chung‐Ang University College of Medicine Gwangmyeong‐si Korea; ^2^ Department of Dermatology Chung‐Ang University Hospital, Chung‐Ang University College of Medicine Seoul Korea; ^3^ Department of Dermatology Konkuk University School of Medicine Seoul Korea; ^4^ Department of Dermatology Kangbuk Samsung Hospital, Sungkyunkwan University School of Medicine Seoul Korea

**Keywords:** animal‐origin free, Asian, botulinum toxin, glabellar lines, human serum albumin free

## Abstract

**Background:**

Botulinum toxin is a key treatment for dynamic wrinkles.

**Objective:**

This study evaluates CKDB‐501A, a botulinum toxin completely free from animal‐derived components including human‐serum albumin, comparing its efficacy and safety to onabotulinumtoxinA (ONA) for the treatment of moderate‐to‐severe glabellar lines.

**Methods:**

In this phase 3 trial, 300 subjects with moderate‐to‐severe glabellar lines were randomized to receive CKDB‐501A or ONA. The primary efficacy endpoint was the investigator‐assessed improvement rate for frowning at week 4, defined as a ≥ 2‐point improvement from baseline on the 4‐point Facial Wrinkle Scale (FWS). Secondary efficacy endpoints included photo‐assessed improvement rates and subjects' overall assessment and satisfaction. Safety was evaluated by the monitoring of adverse events (AEs) and neutralizing antibodies formation.

**Results:**

At week 4, 80.69% of the CKDB‐501A group achieved a ≥ 2‐point improvement in FWS score versus 70.83% for ONA, confirming non‐inferiority (95% CI: 0.09–19.55, *p* = 0.0491). Secondary endpoints showed no significant differences between groups, with sustained efficacy up to 16 weeks. Approximately 70% maintained at least a 1‐point improvement. Photo‐assessed and subjects' overall improvement and satisfaction rates were consistent with primary findings. Both treatments had comparable safety profiles, with no AEs related to the local and distant spread of toxin, hypersensitivity reactions, or neutralizing antibodies formation.

**Conclusion:**

CKDB‐501A is a safe and effective alternative to existing botulinum toxin products for treating moderate‐to‐severe glabellar lines, offering benefits of improved biocompatibility and reduced risk of allergic reactions.

Clinicaltrials.gov: NCT05804656.

## 1 Introduction

Botulinum toxin, produced by *Clostridium botulinum* , has revolutionized cosmetic dermatology. Initially used therapeutically in neurology, its cosmetic potential was later explored, leading to several commercially available formulations [1]. These are primarily used for dynamic wrinkles, particularly glabellar lines, the vertical frown lines between the eyebrows. The most well‐known botulinum toxin products include onabotulinumtoxinA (ONA: Botox, AbbVie/Allergan, Irvine, CA, USA), abobotulinumtoxinA (ABO: Dysport, Ipsen, Les Ulis, France) and incobotulinumtoxinA (INCO: Xeomin, Merz Pharmaceuticals GmbH, Frankfurt, Germany). While derived from the same neurotoxin, each product has distinct formulations, dosing guidelines, and clinical efficacies tailored for cosmetic use [1, 2].

ONA, the first botulinum toxin A approved by the Food and Drug Administration (FDA) for cosmetic use in 2002, remains a cornerstone treatment for glabellar lines. ABO, approved in 2009, has a slightly different diffusion profile and dosing units. INCO, introduced in 2010, lacks complexing proteins, potentially reducing the risk of antibody formation [1, 2].

Despite their widespread use, these products have certain limitations. Specifically, they rely on animal‐derived components, such as human‐serum albumin (HSA), as stabilizers, which are crucial for maintaining product stability but raise concerns regarding potential allergic reactions and the development of neutralizing antibodies. Additionally, variability in diffusion profiles and the potential for adverse events related to the local and distant spread of toxin effect, such as ptosis, also highlight the need for continuous improvement in this field [2].

CKDB‐501A (Tyemvers, Chong Kun Dang BiO Corporation, Seoul, Korea) is a new animal‐origin‐free botulinum toxin A product, formulated as a vacuum‐dried powder. It is produced from a *Clostridium botulinum* Type A X58540 isolated from cattle feces (GenBank: CP068960.1) and features a neurotoxin protein complex with a molecular weight of about 900 kDa, containing a neurotoxin part which has the same amino acid sequence as ONA [3]. CKDB‐501A addresses safety concerns, including infection risks from blood‐borne pathogens and Transmissible Spongiform Encephalopathy (TSE). By eliminating animal‐derived components from its manufacturing process entirely, CKDB‐501A aims to offer a more biocompatible and ethically sound option for patients seeking cosmetic treatments.

The safety and efficacy of CKDB‐501A were evaluated in a phase 1 study (NCT05292638), with a phase 3 trial planned to demonstrate its non‐inferiority to ONA in treating moderate‐to‐severe glabellar lines.

## 2 Methods and Materials

This randomized, double‐blind, single‐treatment, non‐inferiority phase 3 trial was conducted at three sites in South Korea. Institutional review boards approved the protocols. The study adhered to the protocols, Helsinki Declaration and International Council for Harmonization Good Clinical Practice guidelines, with written consent obtained from all subjects.

Adults aged 19–65 years with moderate‐to‐severe glabellar lines at maximum frown, assessed by the investigator using the 4‐point Facial Wrinkle Scale (FWS) were eligible. Exclusion criteria included: (1) pregnancy or breastfeeding, (2) neuromuscular disorders such as myasthenia gravis, Lambert‐Easton syndrome, or amyotrophic lateral sclerosis, (3) history of facial nerve paralysis or ptosis, (4) significant facial asymmetry, (5) history of surgery potentially altering facial anatomy (6) skin abnormalities, (7) previous soft tissue augmentation with hyaluronic acid or collagen fillers, lifting laser, or dermal regeneration therapy within 24 weeks prior to screening, (8) previous soft tissue augmentation with semi‐permanent fillers, fat transplantation, or thread lifting with 48 weeks prior to screening, (9) previous treatment with any serotype of botulinum toxin within 24 weeks prior to screening, (10) treatment with muscle relaxant effects within 4 weeks prior to screening, and (11) known allergy or hypersensitivity reaction to the investigational product or its components.

Eligible subjects were randomized 1:1 into CKDB‐501A or ONA groups using interactive web response system. The randomization schedule was generated by an independent biostatistician, ensuring unbiased allocation across treatment groups, which were prospectively stratified by study site. The investigational product (IP) was prepared with preservative‐free sterile saline to 100 U/2.5 mL (4 U/0.1 mL). The prepared injection syringe was then provided to the investigator in a blinded manner. 4 U/0.1 mL of the IP was injected intramuscularly into glabellar lines in total with 2 sites in each corrugator muscle, and 1 site in the procerus muscle for a total dose of 20 U/0.5 mL. Follow‐up visits occurred every 4 weeks for 16 weeks.

### 2.1 Efficacy Assessment

The primary endpoint was the proportion of subjects achieving at least a 2‐point improvement in the investigator‐assessed FWS score for frowning from baseline at 4 weeks post‐administration. Secondary endpoints included achieving at least a 2‐point improvement in the investigator‐assessed FWS score for frowning at weeks 8, 12, and 16, and achieving at least a 1‐point improvement in the investigator‐assessed FWS score for frowning and at rest at these time points. Three independent evaluators assessed subjects' FWS scores using standardized digital photographs at every visit. The proportion of subjects achieving at least a 2‐point improvement in photo‐assessed FWS score for frowning from baseline at weeks 4, 8, 12, and 16 was analyzed as secondary endpoint. At each post‐treatment visit, subjects assessed their overall improvement and satisfaction scores for glabellar lines. The efficacy was evaluated based on the proportion of subjects achieving an overall improvement score of +2 points or higher (indicating more than moderate improvement), and those achieving a satisfaction score of 6 (satisfied) or 7 (very satisfied). The efficacy measurements used in the study are summarized in Table S1.

To support the primary endpoint, a post hoc analysis was conducted, including subgroup analyses based on baseline severity of glabellar lines (moderate, severe), age groups (19–39, 40–49, 50–65 years), and gender. Additionally, the analysis examined the proportion of subjects achieving an investigator‐assessed FWS score of 0 (none) at maximum frown at week 4.

### 2.2 Safety Assessment

Safety endpoints included physical examinations, laboratory tests, vital signs, and adverse events (AEs). AEs related to local and distant spread of toxin were predefined as adverse events of special interest (AESI) following the FDA guidance [4], and acute AEs occurring within 30 min post‐administration were closely monitored. Subjects were observed for signs of both immediate and delayed hypersensitivity reactions including urticaria, angioedema, anaphylaxis, or delayed rash, during a 30‐min post‐administration observation period and at each scheduled follow‐up visit. AEs were coded using preferred terms according to the Medical Dictionary for Regulatory Activities (MedDRA, version 26.1). Immunogenicity was evaluated at baseline and 16 weeks post‐administration by assessing the formation of neutralizing antibodies (Nabs) [5]. Blood samples were collected at both time points and analyzed using a mouse protection assay, historically regarded as the gold standard for Nab detection and quantification. In this assay, each subject's serum was incubated with a standardized amount of botulinum neurotoxin and injected intraperitoneally into four mice. A positive result was defined as the survival of at least three out of four mice, indicating the presence of neutralizing antibodies in the serum.

### 2.3 Statistical Analysis

The sample size was estimated based on the primary efficacy endpoint, using a non‐inferiority margin of −15% derived from previous studies [6, 7, 8]. This margin aligns with prior botulinum toxin studies in aesthetic indications, where non‐inferiority margins between −10% and −15% have been widely accepted in global studies. In particular, the Korean Ministry of Food and Drug Safety (MFDS) has adopted a −15% non‐inferiority margin as an acceptable threshold for evaluating comparative efficacy. This threshold is considered clinically acceptable, ensuring that the new product maintains a level of effectiveness that supports patient satisfaction and therapeutic viability. To achieve 80% power at a significance level (*α*) of 0.05 for a two‐sided test, 300 subjects (150 per group) were required, assuming a 10% dropout rate. The difference between treatment groups for the primary efficacy endpoint was analyzed using the Cochran–Mantel–Haenszel (CMH) test, adjusting for study site. Non‐inferiority of CKDB‐501A to ONA was concluded if the lower limit of the 95% Confidence Interval (CI) exceeded the predefined margin of −15%. Secondary efficacy endpoints, specifically investigator‐assessed FWS score, were also evaluated using the CMH test. Pearson's chi‐square test or Fisher's exact test was used to analyze the other efficacy and safety endpoints, as well as in the post hoc analysis. Statistical analyses were conducted using SAS (version 9.4, SAS Institute, Cary, NC, USA).

Statistical analyses followed the intent‐to‐treat principle. Safety analyses were conducted on the Safety Analysis Set (SAS), including all subjects who received the IP as treated. Efficacy analyses were performed on both the Full Analysis Set (FAS) and the Per‐Protocol Set (PPS), with the PPS being primary. The PPS included FAS subjects who adhered to the protocol without major deviations. Missing data in the FAS efficacy analysis were handled using the Baseline Observation Carried Forward method, while no imputation was applied in the PPS efficacy and SAS safety analyses.

## 3 Results

### 3.1 Subject Disposition and Baseline Characteristics

**FIGURE 1:**
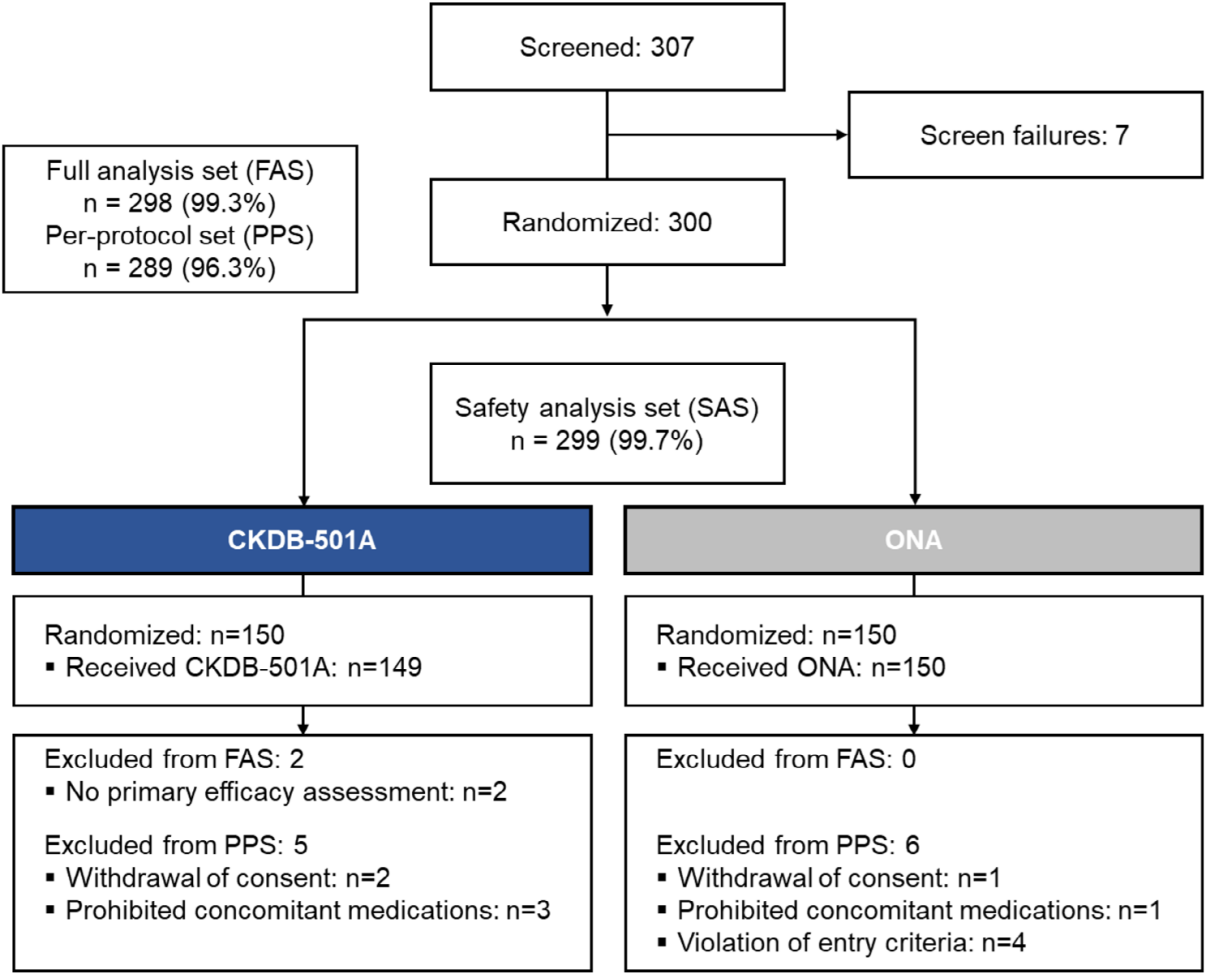
Subject disposition.

Between April and July 2023, 300 subjects were randomized from 307 screened. Among them, 299 were treated and included in the SAS (CKDB‐501A: *n* = 149, ONA: *n* = 150), 298 in the FAS (CKDB‐501A: *n* = 148, ONA: *n* = 150), and 289 in the PPS (CKDB‐501A: *n* = 145, ONA: *n* = 144) (Figure 1). The baseline characteristics were well‐balanced between groups (Table 1). The mean age (years) was 46.80 ± 8.39 in the CKDB‐501A group and 47.33 ± 8.00 in the ONA group, showing no significant between‐group difference (*p* = 0.5727). Gender distribution showed 28.38% males and 71.62% females in the CKDB‐501A group, and 24.67% males and 75.33% females in the ONA group, indicating a higher proportion of females in both groups without significant between‐group difference (*p* = 0.4680). The baseline severity was also similar between groups, with 64.86% of subjects in the CKDB‐501A group and 65.33% in the ONA group having an investigator‐assessed FWS score of 2 (moderate) at maximum frown (*p* = 0.9324).

**TABLE 1:** Demographics and baseline characteristics (FAS).

	CKDB‐501A (*n* = 148)	ONA (*n* = 150)	*p*
Age, years, mean ± SD	46.80 ± 8.39	47.33 ± 8.00	0.5727^T^
Age group, *n* (%)			0.4819^C^
19–29 years	3 (2.03)	1 (0.67)	
30–39 years	24 (16.22)	22 (14.67)	
40–49 years	71 (47.97)	69 (46.00)	
50–59 years	35 (23.65)	47 (31.33)	
60–65 years	15 (10.14)	11 (7.33)	
Gender, *n* (%)			0.4680^C^
Male	42 (28.38)	37 (24.67)	
Female	106 (71.62)	113 (75.33)	
Investigator‐assessed FWS scores at maximum frown, *n* (%)			0.9324^C^
2 (Moderate)	96 (64.86)	98 (65.33)	
3 (Severe)	52 (35.14)	52 (34.67)	
History of botulinum toxin use	92 (62.16)	93 (62.00)	0.9770^C^

*Note:* Data are presented as mean ± standard deviation (SD) or number (%) as appropriate. Statistical analysis included the independent *t*‐test (T) or Wilcoxon rank‐sum test (W) for continuous variables, depending on data normality, and chi‐squared test (C) or Fisher's exact test (F) for categorical variables.

Abbreviations: FAS, full analysis set; FWS, facial wrinkle scale; ONA, onabotulinumtoxinA.

### 3.2 Primary Efficacy Endpoint

**FIGURE 2:**
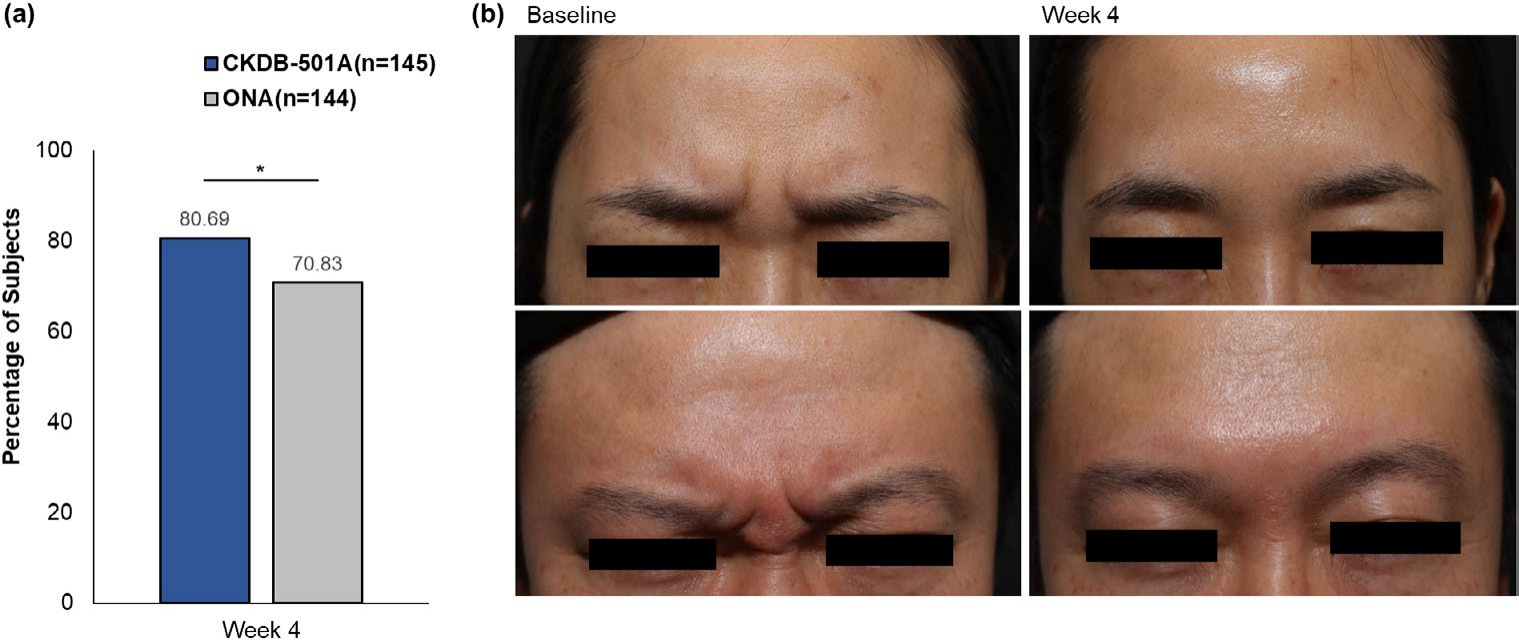
Primary efficacy results (a) investigator‐assessed ≥ 2‐point improvement rates. (b) Photographs of female (upper) and male (lower) subjects treated with CKDB‐501A achieving ≥ 2‐point improvement. The primary efficacy endpoint was defined as the subject achieving at least a 2‐point improvement from baseline in the investigator‐assessed FWS score at maximum frown. Photographs of subjects treated with CKDB‐501A who achieved a ≥ 2‐point improvement are shown in (b). Left photographs show the baseline condition, and right photographs show the condition at week 4. Subjects fully consented to the use of their photographs for publication purposes. Statistical analysis was conducted on the per‐protocol set using the Cochran–Mantel–Haenszel test, adjusting for study site. An asterisk (*) indicates a significant difference at *p* < 0.05. ONA, onabotulinumtoxinA.

At week 4, the investigator‐assessed ≥ 2‐point improvement rate for frowning was 80.69% in the CKDB‐501A group and 70.83% in the ONA group within the PPS population (Figure 2a,b). The improvement rate was statistically significantly higher in the CKDB‐501A group (*p* = 0.0491). The between‐group difference was 9.82%, and the lower limit of the two‐sided 95% CI was 0.09%, exceeding the non‐inferiority margin of −15%, thus demonstrating the non‐inferiority of CKDB‐501A to ONA (Table 2).

**TABLE 2:** Investigator‐assessed ≥ 2‐point improvement rate at maximum frown.

	CKDB‐501A	ONA	Difference [95% CI]	*p*
PPS population (primary)				
Week 4, % (*n*)	80.69 (117/145)	70.83 (102/144)	9.82 [0.09, 19.55]	0.0491
Week 8, % (*n*)	47.59 (69/145)	46.85 (67/143)	0.69 [−10.76, 12.13]	0.9063
Week 12, % (*n*)	15.28 (22/144)	19.44 (28/144)	−4.07 [−12.73, 4.60]	0.3597
Week 16, % (*n*)	3.47 (5/144)	4.17 (6/144)	−0.70 [−5.12, 3.71]	0.7567
FAS population				
Week 4, % (*n*)	79.05 (117/148)	70.67 (106/150)	8.49 [−1.22, 18.20]	0.0877
Week 8, % (*n*)	46.62 (69/148)	46.00 (69/150)	0.64 [−10.59, 11.88]	0.9105
Week 12, % (*n*)	14.86 (22/148)	19.33 (29/150)	−4.26 [−12.71, 4.20]	0.3259
Week 16, % (*n*)	3.38 (5/148)	4.00 (6/150)	−0.65 [−4.92, 3.62]	0.7666

*Note:* The PPS comprised subjects from the FAS who completed the study without major protocol deviations. The FAS included subjects who received the investigational product after randomization and had at least one data point for the primary efficacy assessment. Statistical analysis employed Cochran‐Mantel‐Haenszel test, adjusting for study site, to determine *p*‐values.

Abbreviations: CI, confidence interval; FAS, full analysis set; ONA, onabotulinumtoxinA; PPS, per‐protocol set.

### 3.3 Secondary Efficacy Endpoint

**FIGURE 3:**
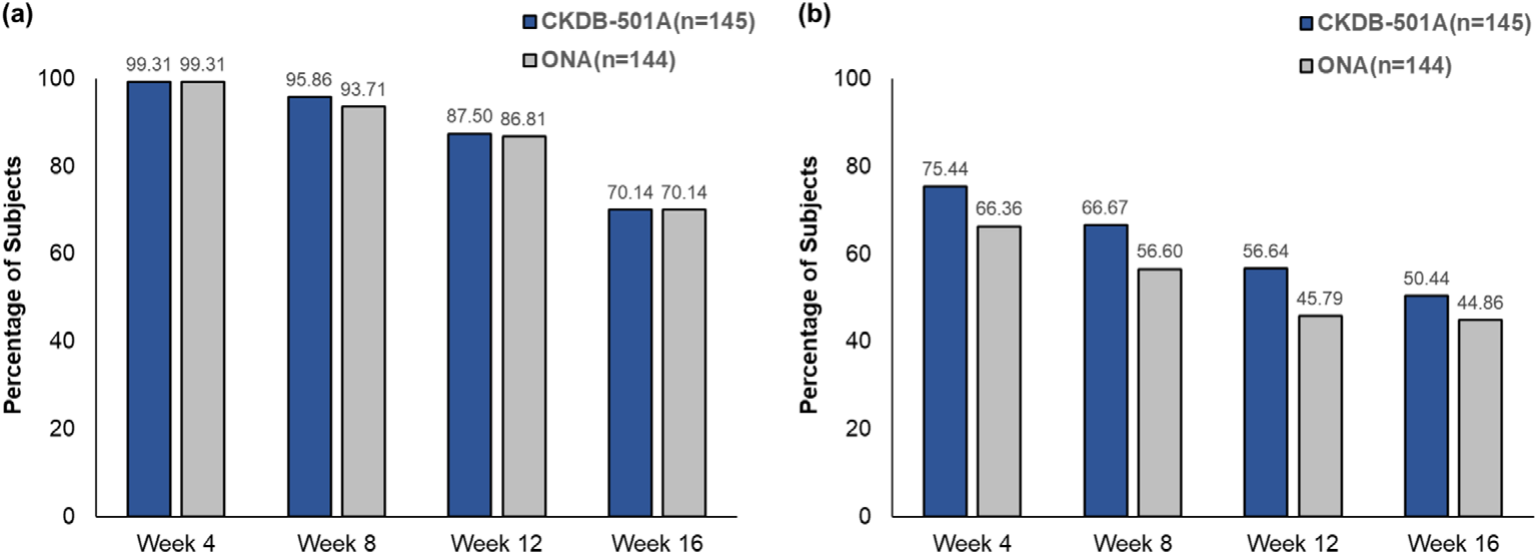
Investigator‐assessed ≥ 1‐point improvement rates (a) at maximum frown (b) at rest. Statistical analysis was conducted on the per‐protocol set using the Cochran–Mantel–Haenszel test, adjusting for study site. When analyzing ≥ 1‐point improvement at rest, subjects with a baseline rest score of 0 were excluded. ONA, onabotulinumtoxinA.

No significant between‐group differences in the investigator‐assessed ≥ 2‐point improvement rate for frowning were observed at 8, 12, and 16 weeks (*p* > 0.05). The lower limits of the two‐sided 95% CI for the differences also exceeded the non‐inferiority margin of −15% at all time points, confirming that the glabellar line improvement effect of CKDB‐501A was non‐inferior to ONA up to 16 weeks post‐administration (Table 2). Positive outcomes were prominently evident when defining the improvement rate for frowning as at least a 1‐point improvement from baseline. Both groups exhibited the improvement rate of approximately 99% at 4 weeks, declining to 70% by 16 weeks (Figure 3a).

For the investigator‐assessed ≥ 1‐point improvement rate at rest, the CKDB‐501A group consistently showed a numerically higher improvement rate compared to the ONA group at all time points, though no significant between‐group differences observed (*p* > 0.05) (Figure 3b).

At week 4, the photo‐assessed ≥ 2‐point improvement rate for frowning in the CKDB‐501A and ONA groups of the PPS population was 88.97% and 82.64%, respectively, with a numerically higher improvement rate observed in the CKDB‐501A group. However, there were no significant between‐group differences at any time point (*p* > 0.05) (Table 3).

**TABLE 3:** Photo‐Assessed ≥ 2‐point improvement rate at maximum frown.

	CKDB‐501A	ONA	Difference [95% CI]	*p*
PPS population (primary)				
Week 4, % (*n*)	88.97 (129/145)	82.64 (119/144)	6.33 [−1.69, 14.34]	0.1233^C^
Week 8, % (*n*)	67.59 (98/145)	58.74 (84/143)	8.84 [−2.25, 19.94]	0.1197^C^
Week 12, % (*n*)	27.08 (39/144)	29.86 (43/144)	‐2.78 [−13.20, 7.64]	0.6015^C^
Week 16, % (*n*)	15.28 (22/144)	18.75 (27/144)	3.47 [−12.14, 5.20]	0.4330^C^
FAS population				
Week 4, % (*n*)	88.51 (131/148)	82.00 (123/150)	6.51 [−1.50, 14.53]	0.1130^C^
Week 8, % (*n*)	67.57 (100/148)	58.00 (87/150)	9.57 [−1.35, 20.49]	0.0876^C^
Week 12, % (*n*)	27.03 (40/148)	29.33 (44/150)	‐2.31 [−12.52, 7.91]	0.6582^C^
Week 16, % (*n*)	14.86 (22/148)	18.00 (27/150)	‐3.14 [−11.54, 5.27]	0.4654^C^

*Note:* Three independent evaluators, who were familiar with the glabellar line photographic guideline and qualified to assess glabellar lines, evaluated the subject's glabellar line severity using the 4‐point FWS based on photographs. To eliminate timing bias, photographs were randomly arranged without time point identification. Evaluators independently assessed the photographs, and the final grade was determined if at least two evaluators agreed. Statistical analysis included the chi‐squared test (C) or Fisher's exact test (F) to determine *p*‐values.

Abbreviations: CI, confidence interval; FAS, full analysis set; FWS, facial wrinkle scale; ONA, onabotulinumtoxinA; PPS, per‐protocol set.

Consistent results were also observed in both subjects' overall improvement and satisfaction rates, underscoring the clinical benefit of CKDB‐501A comparable to ONA (Table S2).

### 3.4 Post Hoc Analyses

**FIGURE 4:**
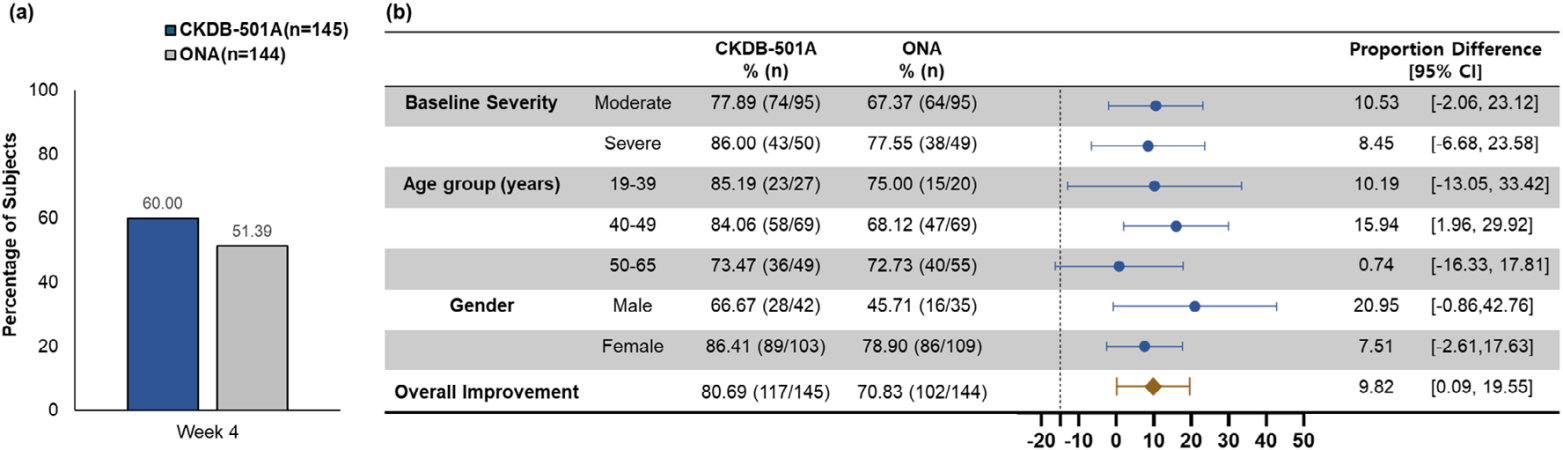
Post hoc analysis results (a) proportion of subjects achieving an investigator‐assessed FWS score of 0 (none) at maximum frown at week 4, (b) forest plot for investigator‐assessed ≥ 2‐point improvement rate at maximum frown at week 4 by baseline severity, age group, and gender. Statistical analysis was conducted on the per‐protocol set using the chi‐squared test (C) or Fisher's exact test (F). A vertical dotted line indicates a non‐inferiority margin of −15%. CI, confidence interval; ONA, onabotulinumtoxinA.

In the post hoc analysis, 60.00% of subjects treated with CKDB‐501A achieved an investigator‐assessed FWS score of 0 at maximum frown at week 4, compared to 51.39% with ONA (Figure 4a).

The results of subgroup analysis for the investigator assessed ≥ 2‐point improvement rates for frowning at week 4 by baseline severity, age groups, and gender are presented in Figure 4b.

When stratified by baseline severity, subjects with moderate frown lines achieved improvement rates of 77.89% in the CKDB‐501A group and 67.37% in the ONA group, while those with severe frown lines had improvement rates of 86.00% and 77.55%, respectively. Although there was a trend towards slightly higher improvement in subjects with severe frown lines, no statistically significant differences found. Across age group (19–39, 40–49, and 50–65 years), both treatment groups exhibited consistent improvement rate. Regarding gender, females generally exhibited greater improvement in both treatment groups. Specifically, females in the CKDB‐501A group showed the improvement rate of 86.41% compared to 78.90% in the ONA group, whereas males had improvement rates of 66.67% and 45.71%, respectively.

Post hoc analysis results were consistent with the primary efficacy endpoints, confirming the reliability of our study's findings.

### 3.5 Safety Analyses

A total of 39 subjects experienced AEs, with ‘blood cholesterol increased’ and ‘acne’ being the most frequent in both groups (Table S3). Most AEs were mild or moderate in severity. The incidence of adverse drug reaction (ADR) was 0.67% in the CKDB‐501A group and no ADR was reported in the ONA group, with no between‐group difference (*p* = 0.4983). The reported ADR was ‘genital rash’, assessed as ‘unassessable/unclassifiable’. One subject treated with CKDB‐501A experienced a serious AE, specifically ‘Femoral neck fracture’, assessed as ‘not related’ to the IP. There were no reports of acute AEs, serious ADRs, AESI, or formation of Nabs in both groups. All subjects tested negative for neutralizing antibodies at both baseline and week 16, indicating no detectable immunogenic response to either CKDB‐501A or ONA after a single administration. No immediate or delayed hypersensitivity reactions were reported in either treatment group throughout the study. No AEs led to temporary or permanent termination of the IP, and no subjects discontinued the study due to AEs.

## 4 Discussion

This study demonstrated that CKDB‐501A is effective and safe for the treatment of moderate‐to‐severe glabellar lines. The newly developed product showed comparable efficacy to ONA, with a similar safety profile. Our findings align with previous studies that have established botulinum toxin formulations as effective treatments for glabellar lines [6, 7, 8, 9, 10, 11, 12]. However, CKDB‐501A's unique formulation, which utilize sucrose as a stabilizer to eliminate animal‐derived components, including HSA, from its manufacturing process entirely, distinguishes it from traditional botulinum toxin products. Historically, some botulinum toxin formulations included stabilizers derived from animal sources, such as bovine gelatin, posing concerns about potential immunogenicity and allergic reactions. Modern formulations like ONA, ABO, and INCO predominantly use HSA [1, 2]. While these stabilizers have improved safety profiles compared to animal‐derived components, they are not entirely risk‐free. HSA, despite being human‐derived, may still trigger allergic reactions in rare cases. Additionally, some formulations utilize polysorbates 20 and 80 as surfactants, which have been associated with their own set of adverse reactions, such as hypersensitivity and anaphylaxis [13].

The introduction of CKDB‐501A provides clinicians with a new option that combines efficacy with improved biocompatibility. This product is particularly beneficial for patients who are concerned about animal‐derived ingredients or who have experienced allergic reactions with other botulinum toxin products.

In this study, CKDB‐501A was compared to ONA in improving the glabellar lines severity score, representing FWS score. At week 4, the investigator‐assessed improvement rate for frowning (defined as ≥ 2‐point improvement from baseline) was statistically significantly higher with CKDB‐501A compared to ONA (80.69% vs. 70.83%, *p* = 0.0491), with maintaining the non‐inferiority up to 16 weeks. The positive outcomes were prominently evident when defining the improvement for frowning as at least a 1‐point decrease from baseline. Approximately 70% of subjects maintained the improvement until week 16. Consistent results from secondary efficacy endpoints further supported the robustness of these findings.

A recent network meta‐analysis of 4706 patients across 18 randomized controlled trials assessed various botulinum toxin formulations for their efficacy in the treatment of moderate‐to‐severe glabellar lines [14]. According to the analysis, daxibotulinumtoxinA (DAXI) achieved the highest surface under the cumulative ranking curves (SUCRA) probability value at 86.2%, indicating its strong likelihood of achieving a ≥ 2‐point improvement at 1 month at maximum frown. Following DAXI, ONA ranked second with a SUCRA value of 72.3%, INCO at 63.1%, liquid formulation of ABO at 50.2%, and ABO at 36.0%.

Notably, our study comparing CKDB‐501A to ONA found CKDB‐501A to have a statistically significantly higher improvement rate at week 4 (80.69% vs. 70.83%, *p* = 0.0491). Additionally, post hoc analyses revealed that 60.00% of subjects treated with CKDB‐501A achieved an investigator‐assessed FWS score of 0 (none), compared to 51.39% with ONA. These results underscore CKDB‐501A as a promising new treatment option, potentially offering efficacy comparable to or even more effective than ONA and possibly others. However, further research is essential to validate these initial findings.

Subgroup analyses for the primary efficacy endpoint were conducted to explore the consistency of efficacy across different demographics. Subjects with both moderate and severe frown lines showed significant improvement following treatment. This consistency across severity levels supports the broad applicability of CKDB‐501A. The improvement was consistently observed across different age groups (19–39, 40–49, and 50–65 years), underscoring CKDB‐501A as a versatile treatment option suitable for adults of all ages. For gender, both males and females showed significant improvement, however, females exhibited a tendency towards a higher improvement rate compared to males. This observation aligns with published studies suggesting that botulinum toxin may exhibit varying efficacy between genders due to differences in skeletal muscle mass [15]. Females typically have lower muscle mass in the glabellar region compared to males, potentially leading to a more pronounced effect of the toxin. It's important to interpret these findings cautiously due to the relatively small sample sizes within each subgroup.

Reports indicated that the formation of NAbs against botulinum toxin type A is relatively rare but can occur, potentially diminishing the clinical effectiveness and leading to secondary non‐responsiveness in some patients. A comprehensive meta‐analysis of over 5800 subjects treated with ONA across multiple indications found only 0.5% developed NAbs, with 0.3% remaining NAb‐positive at the end of the study. Importantly, the formation of NAbs did not consistently correlate with clinical response failure, suggesting other factors may also contribute to treatment outcomes. Furthermore, case studies have documented instances where patients developed high titers of NAbs after repeated treatments, resulting in reduced or no clinical effect. These cases highlight the impact of factors such as the total cumulative dose and frequency of exposure to the toxin on the likelihood of developing NAbs [16, 17, 18].

In this study, both CKDB‐501A and ONA showed negative results for NAbs before and after treatment. Although the study evaluated a single treatment and did not assess repeated treatments or cumulative doses, the results indicate that NAbs did not affect the observed efficacy.

Regarding safety, CKDB‐501A exhibited a favorable profile. The AEs reported were mostly mild, comparable to those observed with ONA. Importantly, there were no cases of hypersensitivity reactions or AEs related to the local or distant spread of the toxin effect, such as ptosis, muscle weakness, or dysphagia. The absence of NAbs in any subjects indicates a low potential for immunogenicity, which is a crucial factor for repeated treatments.

In conclusion, CKDB‐501A demonstrated non‐inferiority to ONA in improving the moderate‐to‐severe glabellar lines, with a favorable safety profile. The unique formulation of CKDB‐501A, devoid of animal‐derived stabilizers, addresses potential immunogenicity and allergic reaction concerns, making it a promising option for cosmetic treatments.

While this study demonstrates promising results for CKDB‐501A, it is not without limitations. The study duration was limited to short‐term follow‐up, and it was conducted exclusively on Asian patients, which may limit the generalizability of the findings to other racial groups. Further research is needed to confirm the long‐term safety and efficacy of CKDB‐501A. Larger, multicenter studies with more diverse populations and extended follow‐up periods would provide more comprehensive data.

## Author Contributions

The authors contributed equally to this work.

## Ethics Statement

The study was approved by the institutional review board at each study site (Chung‐Ang University Hospital, 2210‐013‐527, approved on 27 October 2022; Konkuk University School of Medicine, KUMC 2022‐10‐022, approved on 30 November 2022; Kangbuk Samsung Hospital, KBSMC 2022‐11‐012, approved on 14 December 2022). The participants in this manuscript signed a photo release consent form authorizing the reproduction and distribution of any images collected during the study.

## Conflicts of Interest

The authors declare no conflicts of interest.

## Supporting information


**Table S1:** Efficacy measurement.
**Table S2:** Subjects’ overall improvement and satisfaction rates (PPS). Subjects’ overall improvement in glabellar lines were evaluated on a 9‐point scale, with improvement defined as a score of at least +2 points. Subjects’ satisfaction was assessed using a 7‐point scale, with satisfaction defined as a score of at least 6 points. Statistical analysis included the chi‐squared test (C) or Fisher’s exact test (F) to determine *p*‐values. ONA, onabotulinumtoxinA; PPS, per‐protocol set; CI, confidence interval.
**Table S3:** Summary of adverse events (SAS) numbers are number of subjects (%), [number of events]. AEs were corded using MedDRA version 26.1. Statistical analysis utilized the chi‐squared test (C) or Fisher’s exact test (F) to determine *p*‐values. SAS, safety analysis set; ONA, onabotulinumtoxinA; TEAE, treatment emergent adverse event; ADR, adverse drug reaction; SAE, serious adverse event; CI, confidence interval.

## Data Availability

The data that support the findings of this study are available from the corresponding author, K.B.J., upon reasonable request.
