# Supplementary material for: Efficacy and Safety of CKDB‐501A in Treating Moderate‐To‐Severe Glabellar Lines: A Randomized, Double‐Blind, Active‐Controlled, Multi‐Center Phase III Trial
Source: J Cosmet Dermatol. 2025 Jun 30;24(7):e70305. doi: 10.1111/jocd.70305 (PMC12207567; doi:10.1111/jocd.70305)
Supplement: Supplementary file 1 — Table S1: Efficacy measurement. Table S2: Subjects’ overall improvement and satisfaction rates (PPS). Subjects’ overall improvement in glabellar lines were evaluated on a 9‐point scale, with improvement defined as a score of at least +2 points. Subjects’ satisfaction was assessed using a 7‐point scale, with satisfaction defined as a score of at least 6 points. Statistical analysis included the chi‐squared test (C) or Fisher’s exact test (F) to determine p‐values. ONA, onabotulinumtoxinA; PPS, per‐protocol set; CI, confidence interval. Table S3: Summary of adverse events (SAS) numbers are number of subjects (%), [number of events]. AEs were corded using MedDRA version 26.1. Statistical analysis utilized the chi‐squared test (C) or Fisher’s exact test (F) to determine p‐values. SAS, safety analysis set; ONA, onabotulinumtoxinA; TEAE, treatment emergent adverse event; ADR, adverse drug reaction; SAE, serious adverse event; CI, confidence interval. [file JOCD-24-e70305-s001.docx]

**Supplementary material**

**Table S1. Efficacy Measurement**

| **Score** | **Rating** | **Description** |
| --- | --- | --- |
| **4-point facial wrinkle scale at maximum frown** | | |
| 0 | None | Lines are not noted. |
| 1 | Mild | Lines are noted. |
| 2 | Moderate | Lines appear clearly formed. The bottoms of the deepest lines are visible from the surface. |
| 3 | Severe | Lines appear clearly formed. The bottoms of the deepest lines are not visible from the surface. |
| **4-point facial wrinkle scale at rest** | | |
| 0 | None | Lines are not noticeable. |
| 1 | Mild | Lines are somewhat noticeable. |
| 2 | Moderate | Lines are noticeable. |
| 3 | Severe | Lines are readily apparent. |
| **Subject’s overall assessment score** | | |
| +4 | Complete improvement | Nearly 100% improvement |
| +3 | Marked improvement | 75% improvement |
| +2 | Moderate improvement | 50% improvement |
| +1 | Slight improvement | 25% improvement |
| 0 | Unchanged | No difference from before |
| -1 | Slight worsening | 25% worsening |
| -2 | Moderate worsening | 50% worsening |
| -3 | Marked worsening | 75% worsening |
| -4 | Very marked worsening | Nearly 100% worsening |
| **Subject’s satisfaction score** | | |
| 1 | Very dissatisfied | - |
| 2 | Dissatisfied | - |
| 3 | Somewhat dissatisfied | - |
| 4 | Indifferent | - |
| 5 | Somewhat satisfied | - |
| 6 | Satisfied | - |
| 7 | Very satisfied | - |

**Table S2. Subjects’ Overall Improvement and Satisfaction Rates (PPS)**

|  | **CKDB-501A** | **ONA** | **Difference**  **[95% CI]** | ***p*-Value** |
| --- | --- | --- | --- | --- |
| **Subjects’ Overall Improvement Rate** | | | | |
| Week 4, % (n) | 93.79  (136/145) | 93.06  (134/144) | 0.74  [-4.98, 6.45] | 0.8003^C^ |
| Week 8, % (n) | 91.72  (133/145) | 90.91  (130/143) | 0.82  [-5.69, 7.32] | 0.8060^C^ |
| Week 12, % (n) | 78.47  (113/144) | 83.33  (120/144) | -4.86  [-13.92, 4.20] | 0.2940^C^ |
| Week 16, % (n) | 68.06  (98/144) | 74.31  (107/144) | -6.25  [-16.69, 4.19] | 0.2416^C^ |
| **Subjects’ Satisfaction Rate** | | | | |
| Week 4, % (n) | 87.59  (127/145) | 88.19  (127/144) | -0.61  [-8.13, 6.91] | 0.8741^C^ |
| Week 8, % (n) | 80.00  (116/145) | 85.31  (122/143) | -5.31  [-14.04, 3.41] | 0.2338^C^ |
| Week 12, % (n) | 73.61  (106/144) | 72.92  (105/144) | 0.69  [-9.53, 10.92] | 0.8941^C^ |
| Week 16, % (n) | 62.50  (90/144) | 70.83  (102/144) | -8.33  [-19.18, 2.51] | 0.1336^C^ |

Subjects' overall improvement in glabellar lines was evaluated on a 9-point scale, with improvement defined as a score of at least +2 points. Subjects' satisfaction was assessed using a 7-point scale, with satisfaction defined as a score of at least 6 points. Statistical analysis included the chi-squared test (C) or Fisher’s exact test (F) to determine p-values. ONA, onabotulinumtoxinA; PPS, per-protocol set; CI, confidence interval.

**Table S3. Summary of Adverse Events (SAS)**

|  | | **CKDB-501A**  **(n=149)** | **ONA**  **(n=150)** | ***p*-Value** |
| --- | --- | --- | --- | --- |
| **TEAE** | | **21 (14.09), [28]** | **18 (12.00), [19]** |  |
|  | 95% CI | [8.94,20.73] | [7.27,18.30] | 0.5909^C^ |
| Common AEs (reported ≥ 1.0% of subjects in any group) | | | | |
|  | Blood cholesterol increased | 6(4.03),[6] | 5(3.33),[5] |  |
|  | Acne | 3(2.01),[4] | 6(4.00),[6] |  |
|  | Alanine aminotransferase increased | 2(1.34),[2] | 0(0.00),[0] |  |
|  | Aspartate aminotransferase increased | 2(1.34),[2] | 0(0.00),[0] |  |
|  | Albuminuria | 2(1.34),[2] | 2(1.33),[2] |  |
|  | Hyperkalaemia | 2(1.34),[2] | 0(0.00),[0] |  |
| **ADR** | | **1(0.67),[1]** | **0(0.00),[0]** |  |
|  | 95% CI | [0.02,3.68] | [0.00,2.43] | 0.4983^F^ |
| All ADRs | | | | |
|  | Genital rash | 1(0.67),[1] | 0(0.00),[0] |  |
| **SAE** | | **1(0.67),[1]** | **0(0.00),[0]** |  |
|  | 95% CI | [0.02,3.68] | [0.00,2.43] | 0.4983^F^ |
| All SAEs | | | | |
|  | Femoral neck fracture | 1(0.67),[1] | 0(0.00),[0] |  |

Numbers are number of subjects (%), [number of events]. AEs were corded using MedDRA version 26.1. Statistical analysis utilized the chi-squared test (C) or Fisher’s exact test (F) to determine p-values. SAS, safety analysis set; ONA, onabotulinumtoxinA; TEAE, treatment emergent adverse event; ADR, adverse drug reaction; SAE, serious adverse event; CI, confidence interval.
